# Enabling Factors for Improved Malaria Chemoprophylaxis Compliance

**DOI:** 10.4269/ajtmh.2012.12-0277c

**Published:** 2012-11-07

**Authors:** Malick Diara, Andry Nowosiwsky, Sonia Harmen, Nicholas Burke, Martin Alilio

**Affiliations:** Global Medicine & Occupational Health Department; Exxon Mobil; Houston, Texas; E-mail: malick.diara@exxonmobil.com; Medicine & Occupational Health Department; Esso Highland Limited; Port Moresby, Papua New Guinea; USAID/Bureau for Global Health; Office of Health, Infectious Disease and Nutrition; Washington, DC

Dear Sir:

The article, “Compliance with Antimalaria Chemoprophylaxis in a Combat Zone,” confirmed the limited adherence to such preventive measures among military service members. The authors analyzed the reasons for noncompliance while presenting measures undertaken to address them. These measures include education and training, supply of medicines before departure, directly observed therapy, and an established discipline policy with consequences rarely applied but supported by the laws of the Uniform Code of Military Justice.^1^

Given the importance of malaria chemoprophylaxis, solutions are needed urgently to address this critical weakness in malaria prevention and control systems, not just for troops but all other non-immune travelers and residents. Poor compliance affects the health of individuals improperly taking the medications. It has the potential to roll back the current gains in malaria control efforts in endemic countries by creating resistance and allowing the re-emergence of parasites in areas that have seen an impressive decline in parasitemia.

For more than a decade, ExxonMobil has assigned thousands of non-immune workers in malaria-endemic countries. They face the same compliance challenges as the United States military service members and other long-term travelers^2^. In 2003, at the inception of our operations in malaria-endemic locations, more than 200 malaria cases were reported. Our strategic response to this was the implementation of a comprehensive malaria program and its integration into the health and safety procedures of our company organizations. As a result, malaria cases decreased to < 15 per year.

The workplace malaria program measures are targeted at individual employees and contractors; they are also embedded into the verification and reporting systems of company operations. They include:

1. A comprehensive awareness program that communicates and confirms the risk of malaria, the program requirements, and each individual's commitment to chemoprophylaxis with their willingness to be tested for compliance verification. Education begins before departure, with consistent reinforcement of key messages upon arrival, during site induction, and safety meetings. Training completion is verified before travel, at arrival in the country, and at specific check points such as company air transportation sites or camp offices. For company providers, malaria program requirements are specified in contractor agreements and subject to review at contractor mobilization.
2. Provision of chemoprophylaxis medicines recommended by the World Health Organization (WHO)^3^ and the U.S. Center for Control of Infectious Diseases.^4^ They include Atovaquone-Proguanil, Mefloquine, and doxycycline, which can be adopted for long-term use,^5^ expand workers' choices, personalize their prescription, enable them to switch medications when needed while representing a panel of medicines with reported equal efficacy and side-effect occurrence.^6^ Medical services are available on site to ensure that medications are tolerated or changed. The costs of medicines are reimbursed by the company for employees and for contractors, through company policies and agreements.
3. Monitoring chemoprophylaxis compliance through random testing of workers. The laboratory test method was developed with a United States-based company.^7^ Between 5,000 and 10,000 tests were performed annually from 2003^8^ to 2011. Non-detect rate of malaria medicines in urine samples tested during this time frame decreased from 2.5% to 0.5%. The urine tests verify the use of malaria chemoprophylaxis, although their protective levels can only be assessed through blood tests. In early 2012, a field test developed with the French Army^9^ for Proguanil and Mefloquine was implemented. It provides results in 10 minutes and enables more practical on-site verification and immediate feedback.
4. A “show-me process” at arrival and departure from work sites. This process requires workers to verify that they are in possession of their malaria chemoprophylaxis and are practicing bite protection (wearing factory-treated permethrin clothing, carrying insect repellent, and documentation of malaria awareness training).
5. Engagement of Senior Management for a malaria control program. Malaria cases, cases of non-detects for urine malaria drug tests, and periodic assessments of the malaria control program elements performed through Operations Integrity Management Systems are reported up line to senior company executives from affiliates. These reports are a part of the site performances in malaria locations. They are stewarded by a Corporate Malaria Steering Committee responsible for guidance and sponsor of program activities. Contractor companies are also assessed and notified of health expectations, including malaria program requirements through various mechanisms before, during, and after mobilization.

Successful programs, including malaria chemoprophylaxis compliance, rely on clear expectations, personal and organizational engagement with effective verification of expectations, and application of corrective action when indicated. Like any behavior required from population groups (observance of speed limits, proper harnessing while working at heights, or adherence to required health practices), repeated training and awareness, individual and leadership commitment, verification of observance, and application of consequences for noncompliance are key enabling factors.
